# Mechanism for Regulation of Melanoma Cell Death via Activation of Thermo-TRPV4 and TRPV2

**DOI:** 10.1155/2019/7362875

**Published:** 2019-02-07

**Authors:** Jiaojiao Zheng, Fangyuan Liu, Sha Du, Mei Li, Tian Wu, Xuejing Tan, Wei Cheng

**Affiliations:** ^1^Institute of Cancer Stem Cell, Dalian Medical University, No. 9 West Section Lvshun South Road, Dalian 116044, China; ^2^The First Affiliated Hospital, Dalian Medical University, No. 222 Zhongshan Road, Dalian 116011, China

## Abstract

**Background:**

Thermo-TRPs (temperature-sensitive transient receptor potential channels) belong to the TRP (transient receptor potential) channel superfamily. Emerging evidence implied that thermo-TRPs have been involved in regulation of cell fate in certain tumors. However, their distribution profiles and roles in melanoma remain incompletely understood.

**Methods:**

Western blot and digital PCR approaches were performed to identify the distribution profiles of six thermo-TRPs. MTT assessment was employed to detect cell viability. Flow cytometry was applied to test cell cycle and apoptosis. Calcium imaging was used to determine the function of channels. Five cell lines, including one normal human primary epidermal melanocytes and two human malignant melanoma (A375, G361) and two human metastatic melanoma (A2058, SK-MEL-3) cell lines, were chosen for this research.

**Results:**

In the present study, six thermo-TRPs including TRPV1/2/3/4, TRPA1, and TRPM8 were examined in human primary melanocytes and melanoma cells. We found that TRPV2/4, TRPA1, and TRPM8 exhibited ectopic distribution both in melanocytes and melanoma cells. Moreover, activation of TRPV2 and TRPV4 could lead to the decline of cell viability for melanoma A2058 and A375 cells. Subsequently, activation of TRPV2 by 2-APB (IC_50_ = 150 *μ*M) induced cell necrosis in A2058 cells, while activation of TRPV4 by GSK1016790A (IC_50_ = 10 nM) enhanced apoptosis of A375 cells. Furthermore, TRPV4 mediated cell apoptosis of melanoma via phosphorylation of AKT and was involved in calcium regulation.

**Conclusion:**

Overall, our studies revealed that TRPV4 and TRPV2 mediated melanoma cell death via channel activation and characterized the mechanism of functional TRPV4 ion channel in regulating AKT pathway driven antitumor process. Thus, they may serve as potential biomarkers for the prognosis and are targeted for the therapeutic use in human melanoma.

## 1. Introduction

Malignant melanoma is a very aggressive and lethal form of cutaneous cancer. It usually derives from the transformation of melanocytes causing dysfunction to normal cellular growth. Increasing studies demonstrated that ion channels play important roles in regulating cellular physiology and pathology in cancers [1–5]. Melanoma cells harboring a diversity of ion channel types have been detected and described [6–8].

Human melanocytes are usually located in the skin and eyes. Several transient receptor potential channels (TRPs) such as TRPA1, TRPV3, TRPV4, TRPM4, and TRPM8 are expressed in the epithelial cells and keratinocytes of the skin [9–11]. Fusi et al. reported that TRPV4 was detected in healthy or inflamed skin and lost both in premalignant lesions and nonmelanoma skin cancer. Selectively reduction of TRPV4 distribution may represent a diagnostic biomarker of skin carcinogenesis [12]. Meanwhile, study suggested that skin cancer may be closely related to TRPV1 and TRPV1 knockout mice were facile to induce skin carcinogenesis [13]. Thus, examination of the distribution pattern and function of TRP ion channels in human melanoma cells is necessary and useful for clinical practice.

Recent studies have found that several TRPs presented in melanoma cells. These TRPs were also involved in regulating cellular functions of human melanoma. In this regard, study identified that TRPM7 could be a protector and detoxifier in both melanocytes and melanoma cells [14]. Further, TRPM8 has been reported to participate in mediating agonist-induced melanoma cell death [15].

Temperature-sensitive transient receptor potential channels (thermo-TRPs) belong to TRP channel superfamily. These channels act as multimode cellular sensors for detecting a variety of environmental stimuli to confer crucial physiological functions, such as thermosensation, chemesthesis, pain and inflammation, and sensation of taste, even a biomarker for certain metastatic cancers [16–23]. Several thermo-TRPs also have been identified to involve in certain types of carcinoma malignancy [24–29]. Mergler et al. reported that functional TRPV1, TRPM8, and TRPA1 were expressed in malignant human uveal melanoma tissues, and some of these channels were also detected in human uveal melanoma cell lines [30]. Another study revealed that overexpression of TRPM2 increased melanoma susceptibility to apoptosis and necrosis [31]. Yamamura et al. found that functional TRPM8 expressed in human melanoma cell line and activation of TRPM8 could inhibit cell viability of human melanoma G361 cells [15]. TRPM8 and TRPA1 channel proteins have been identified to exhibit abnormal expression in melanoma cell lines. Certain compounds such as TRPA1 activators like cinnamaldehyde and allyl isothiocyanate could reduce the proliferation of melanoma cells [32]. Although the existing studies referred to that several thermo-TRPs were distributed in melanoma, the relevant roles have remained essentially unexplored.

In the present study, six thermo-TRPs including four heat sensors of TRPV1/2/3/4 and two cold sensors of TRPA1 and TRPM8 have been investigated among human melanocytes and melanoma cells. Based on current research, our study is to explore the mechanism of ectopic expression of several thermo-TRPs involved in the regulation of cell fate of human melanoma.

## 2. Materials and Methods

### 2.1. Antibodies and Regents

All antibodies to six thermo-TRPs were purchased from Pierce Biotechnology (anti-TRPV1, anti-TRPV2, anti-TRPV3, anti-TRPV4, anti-TRPA1, and anti-TRPM8); other antibodies were obtained from Cell Signaling Technology (CST, anti-AKT, anti-pAKT, anti-P38, anti-pP38, anti-JNK, anti-pJNK, anti-ErK1/2, and anti-pErK1/2). All chemical agents were obtained from Sigma (Merck).

### 2.2. Cell Culture

Human melanoma cell line A375 (malignant, CRL-1619), G361 (malignant, CRL-1424), A2058 (metastatic, CRL-11147), SK-MEL-3 (metastatic, HTB-69), normal human primary epidermal melanocytes (PCS-200-013) were all purchased from American Type Culture Collection (ATCC). A375, A2058 cell lines were cultured in DMEM (ATCC) supplemented with 10% fetal bovine serum (FBS, Gibco). G361, SK-MEL-3 cell lines were cultured in McCoy's 5A (ATCC) supplemented with 10% and 15% fetal bovine serum (FBS, Gibco), respectively. Normal human primary epidermal melanocytes were grown in dermal cell basal media (ATCC, PCS-200-030) supplemented with adult melanocyte growth kit (ATCC, PCS-200-042) components. All cells were grown in a humidified incubator at 37°C, 5% CO_2_ atmosphere. After 1-2 days, cells were chosen to perform calcium imaging or other functional experiments.

### 2.3. Western Blot

Cells at density of 1*∗*10^6^-10^7^ cells/well were rinsed twice with phosphate-buffered saline (PBS) and then lysed on ice with 200 *μ*l cold cell lysis buffer for Western and IP containing 20 mM Tris-HCl (pH = 7.5), 150 mM NaCl, 1% TritonX-100, 2.5 mM sodium pyrophosphate, 1% Na_3_VO_4_, 1 mM EDTA, 0.5 *μ*g/ml leupeptin, and 1 mM phenylmethylsulfonyl fluoride (PMSF) (Beyotime Biotechnology). The lysates were then centrifuged at 14000 g at 4°C for 5 min and the supernatants were stored at -80°C as whole cell protein extracts. Protein concentrations were determined by BCA Protein Assay Kit. Proteins were separated by 8-12% SDS-PAGE gel and then transferred to NC membrane. The NC membrane was blocked with 5% nonfat milk in TBS (10 mM Tris-HCl, pH = 8.0, 150 mM NaCl) for 1 h at room temperature. The NC membrane was then incubated with primary antibody (1:500 dilution) in TBS at 4°C overnight. After incubation with IRDye 800CW Goat Anti-Rabbit IgG (H+L) (1:10000 dilution) in 5% nonfat milk in TBS for 40 min in the dark at room temperature, blots were detected using Odyssey imaging system (Odyssey CLx, LI-COR). All western blots were repeated at least three times for each experiment to confirm the reproducibility of the results.

### 2.4. Validation of Gene Expression by Digital PCR

Total RNA was extracted from cultures of melanocytes and melanoma cell lines using RNAiso plus Kit (TaKaRa). Then, the reverse transcription was performed with PrimeScript RT reagent Kit with gDNA Eraser (TaKaRa). Primer and probe sequences were designed using Primer-Blast of NCBI. To determine the expression profiles of thermo-TRPs in melanoma and melanocytes, digital PCR approach was applied using EvaGreen Kit (Bio-Rad). Primer sequences were given in Table 1. The intact cDNA samples were added to the Bio-Rad 2X ddPCR EvaGreen Supermix at concentration of 50 ng DNA per 20 *μ*l ddPCR reaction. Reaction mixes were thoroughly mixed by brief vortex as avoiding the formation of bubbles, centrifuged for 20 sec and allowed reaction mixes to equilibrate at room temperature for about 3 min, then loaded 20 *μ*l of each reaction mix into a sample well of a DG8 Cartridge for QX200 droplet generator (Bio-Rad) followed by 70 *μ*l of QX200 droplet generation oil for EvaGreen into the oil wells, according to the QX200 droplet generator instruction manual. After droplet generation, droplets were transferred into a clean 96-well plate (twin. tec real-time PCR plates, Eppendorf) and sealed with the PX1PCR plate sealer (Bio-Rad). Then the sealed plate was proceeded to a thermal cycler (T100, Bio-Rad) following standard cycling conditions: 95°C for 5 min, then 40 cycles of 95°C for 30 sec; 60°C for 60 sec, 4°C for 5 min, then 90°C for 5 min, and 4°C infinitely; after thermal cycling, 96-well sealed plate was placed in the QX200 droplet reader (Bio-Rad), which automatically acquired data of the droplets from each well of the plate. Data analysis was performed using QuantaSoft software (Bio-Rad). Using Poisson statistics, the concentration of DNA sample was determined.

### 2.5. Intracellular Calcium Measurement

Calcium imaging was performed to determine intracellular calcium using Fluo-4 Direct Calcium Assay Kit (Invitrogen) according to manufacturer's protocol. Cells at confluence of 40-50% in 35mm culture plate or 6-well plate with 2*∗*10^5^ cells were incubated with Fluo-4 AM (1.25 *μ*M) for 60 min in the dark at room temperature. Intracellular Ca^2+^ signals were acquired by either ImageXpress Micro XL system (Molecular Devices) or living cell imaging system (Leica DMI6000B). The Fluo-4 fluorescence was measured at an excitation wavelength of 488 nm and emission wavelength of 516 nm from the bottom of the plate. Loading and imaging were carried out in Fluo-4 Direct calcium assay buffer at 37°C. Data were then analysed with MetaMorph software (Molecular Devices).

### 2.6. Cell Proliferation Assay (MTT Test)

Cells were cultured in 96-well plate containing a final volume of 200 *μ*l/well at density of 5*∗*10^3^ cells, and then applied compounds to cells and incubated at 37°C for 24 h. The cells were prepared by adding 10 *μ*l 3-(4,5-dimethylthiazol-2-y1)-2,5-diphenyltetrazolium bromide (MTT) solution per well to achieve a final concentration of 0.45 mg/ml with incubation 2-4 h at 37°C, following 100 *μ*l application of solubilization solution to each well to dissolve formazan crystals, mixed to ensure complete solubilization. The quantity of formazan was measured by recording changes in absorbance at 570 nm using a plate reader (EnSpire 2300, PerkinElmer). A reference wavelength of 630 nm was used. The experiments were repeated at least three times.

### 2.7. Apoptosis Detection with Flow Cytometer

A single cell suspension at 1*∗*10^6^ cells/ml washed in phosphate-buffered saline (PBS) was prepared, then centrifuged and resuspended in 10 mM Hepes/NaOH buffer (pH = 7.4). The cells were added FITC-Annexin V to a final concentration of 1 *μ*g/ml, incubated 10 min in the dark at room temperature. Then PI was added to a final concentration of 2 *μ*g/ml, incubated for a further 5 min. Apoptosis was determined by recording right angle and forward light scatter, log green (520 nm) and log red fluorescence (> 650 nm) with flow cytometer (LSRFortessa, Becton Dickinson). Green fluorescence was using the fluorescein filter and a deep red filter was used for red. Care must be taken not to exclude any apoptotic cells. The percentage of apoptotic cells was determined in three independent experiments.

### 2.8. Cell Cycle Analysis by Flow Cytometry

Cells were seeded at density of 1*∗*10^6^ cells/ml and incubated overnight. Then a stain-detergent solution was made up containing 50 *μ*g/ml isotonic propidium iodide (PI) in 0.1% trisodium citrate dihydride with 0.3 *μ*l/ml of Nonidet P-40. Culture medium was removed and cells were rinsed with PBS once, then stain-detergent solution was added and cells were scraped by a rubber policeman, shook vigorously, and dislodged by a fine tipped pipette. Harvested cells were then centrifuged and suspended in fresh stain-detergent solution for flow cytometry (LSRFortessa, Becton Dickinson) analysis with an argon-in laser tuned to 488 nm and measured red fluorescence.

### 2.9. Posttranscriptional Gene Silencing

Gene silencing was used to knockdown targeted proteins of interest. The Dharmacon™ GIPZ™ lentiviral shRNA were purchased from GE which have been bioinformatically verified to match NCBI sequence data. Plasmid was purified using Midi Kit (QIAGEN). The following sequences as sense strand for TRPV2 and TRPV4 were shown as 5′-GCTGAACCTGCTTTACTAT-3′ and 5′-ACCAAGTTTGTTACCAAGA-3′, respectively. Transfections of shRNA were performed using Lipofectamine 3000 (Invitrogen) following manufacturer's instruction. The silencing efficiency was detected by western blot analysis.

### 2.10. Statistical Analysis

The results were expressed as means ± standard error of the mean (SEM). Statistical significance was evaluated by two-tailed student's t-test. All statistical tests were performed via GraphPad Prism 5 as well as Adobe Illustrator CS6. Significance was set at *∗* P < 0.05, *∗∗* P < 0.01, or *∗∗∗* P < 0.001.

## 3. Results

### 3.1. Thermo-TRPs Exhibited Ectopic Expression Pattern in Human Melanoma Cells and Melanocytes

**Figure 1:**
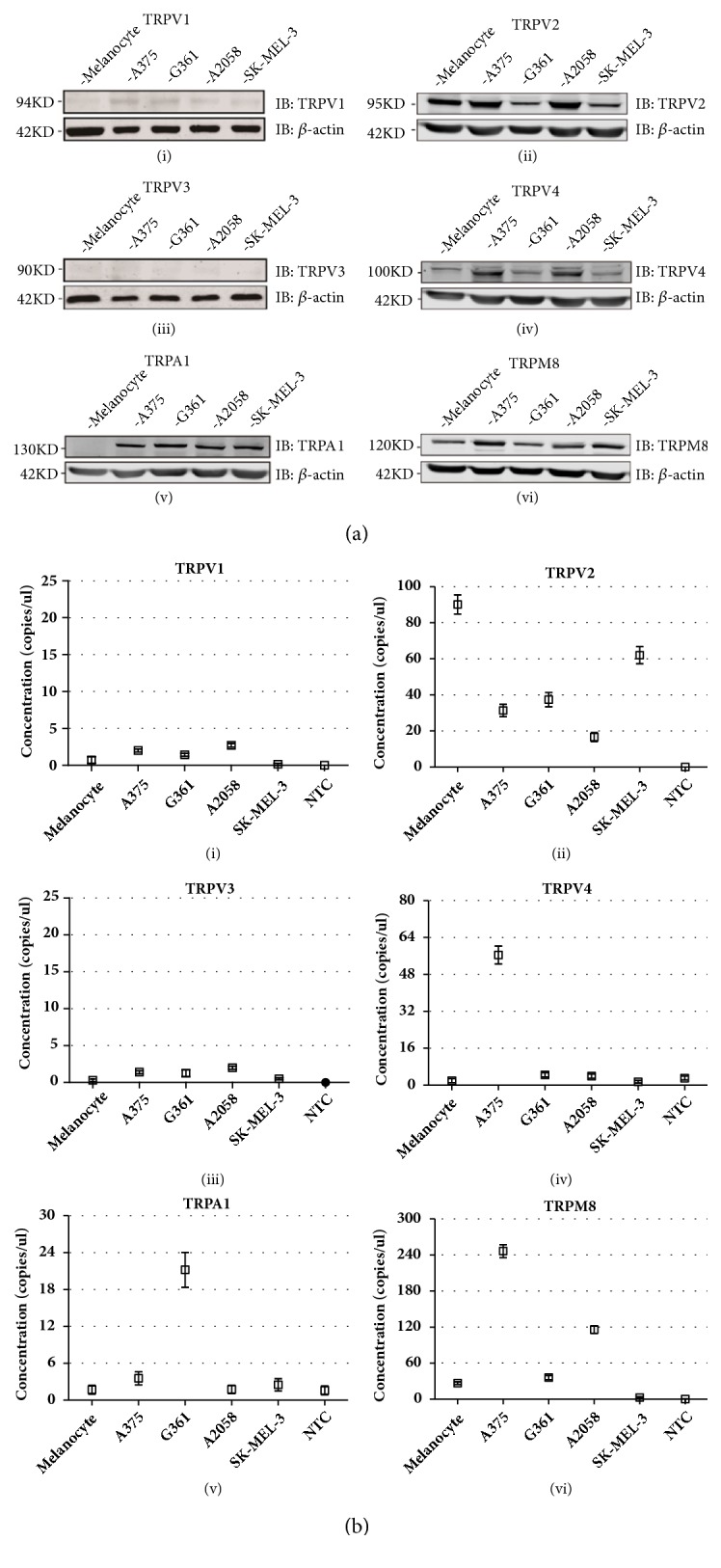
The distribution profiles of six thermo-TRPs in human melanoma cells and melanocytes. (a) Western blot analysis of TRPV1 (i), TRPV2 (ii), TRPV3 (iii), TRPV4 (iv), TRPA1 (v), and TRPM8 (vi) ion channels expression level in protein samples collected from primary epidermal melanocytes, and melanoma cells of A375, G361, A2058, and SK-MEL-3. (b) Droplet digital PCR detection of six thermo-TRPs for TRPV1 (i), TRPV2 (ii), TRPV3 (iii), TRPV4 (iv), TRPA1 (v), and TRPM8 (vi) in primary epidermal melanocytes, and melanoma cells of A375, G361, A2058, and SK-MEL-3. Total mRNA from human primary epidermal melanocytes and melanoma cells of A375, G361, A2058, and SK-MEL-3 were isolated, and digital PCR screening analysis for the indicated genes was performed. Determination of copy numbers per genome of six samples. Concentration values for indicated genes (□). Error bars represented 95% confidence intervals, NTC represented nontemplate control. *β*-actin was used as a positive control, and all tests were performed in at least three independent experiments.

To investigate six thermo-TRPs expression patterns in human melanoma, four melanoma cell lines and primary epidermal melanocytes were chosen for western blot analysis. The assessments clearly showed differential expression profiles of thermo-TRPs, in which TRPV1 was hardly detected in human melanocytes, and very weak expression was found in human melanoma cells (Figure 1(a)(i)). TRPV2 was decreased in G361 and SK-MEL-3 melanoma cells compared to primary epidermal melanocytes (Figure 1(a)(ii)). Neither in melanocytes nor in melanoma cells TRPV3 protein was found (Figure 1(a)(iii)). However, TRPV4 protein was significantly increased in A375 and A2058 cells (Figure 1(a)(iv)). Moreover, previous study has reported that TRPA1 and TRPM8 were expressed in human melanoma [15, 32]; our data showed that TRPA1 protein increased in all four melanoma cells (Figure 1(a)(v)), and TRPM8 protein level was increased in A375 and A2058 cells compared to melanocytes (Figure 1(a)(vi)).

To further confirm the expression profiles of these six thermo-TRPs in melanoma, digital PCR assessment was then conducted and the results showed differential expression pattern of thermo-TRPs in human melanocytes and melanoma cells. Specifically, TRPV1 and TRPV3 transcripts showed very weak expression both in human melanocytes and melanoma cells (Figures 1(b)(i) & 1(b)(iii)) which exhibited good concordance with protein distribution, while TRPV2 was markedly decreased in all four melanoma cell lines compared to melanocytes (Figure 1(b)(ii)), which was discordant with our protein expression results. TRPV4 mRNA was increased significantly in A375 cells compared to melanocytes (Figure 1(b)(iv)). Moreover, TRPA1 showed apparent increase in G361 cells other than melanocytes and other melanoma cells (Figure 1(b)(v)). TRPM8 was found increased in A375 and A2058 cells which was identical with protein expression pattern (Figure 1(b)(vi)).

Because the prior results suggested a discrepancy between protein and mRNA distributions in melanoma, we then examined calcium influx during channel activation and blockade. Calcium imaging indicated that TRPV4 ion channel was functionally expressed in A375 cells, while in A2058 and G361 cells, channel functions were observed inconspicuously (see Figure S1a (i) & (ii)). For TRPV2, both channel common activator of 2-APB (2-aminoethoxydiphenyl borate) and specific agonist of probenecid were inducing similar calcium influx in A2058 cells (Figure S1b (i)), while 2-APB elicited very small calcium influx in G361 cells (Figure S1b (ii)). Our data indicated that both TRPV4 in A375 cells and TRPV2 in A2058 cells might dominate calcium influx during channel activation. But how these two channels function in melanoma remains to be elucidated.

### 3.2. Inhibition of Melanoma Cells Proliferation Modulated by Activation of Thermo-TRPVs

**Figure 2:**
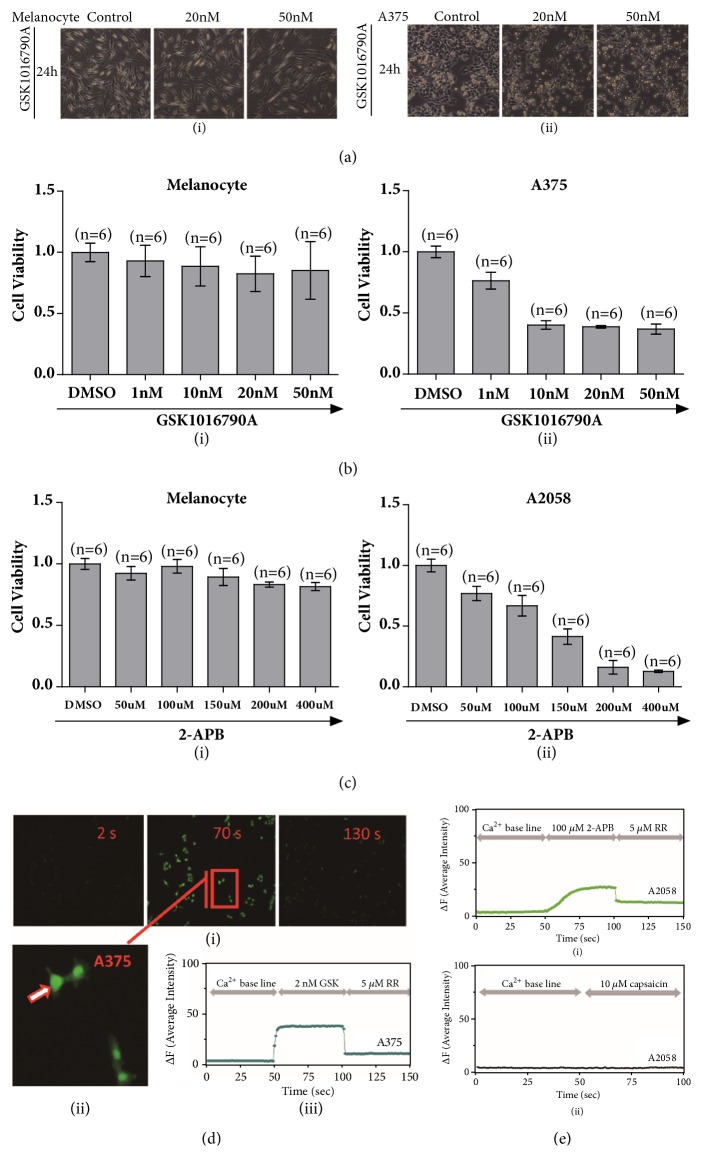
Functional TRPVs mediated proliferation of melanoma cells. (a) Cells morphologically changed after GSK1016790A (20 nM and 50 nM) application to A375 melanoma cells and melanocytes via microscopy imaging (i) & (ii). (b) Cell viability test by MTT experiment exhibited inhibition of cell proliferation for melanoma A375 cells but not for primary epidermal melanocytes by treatment of either DMSO (control) or TRPV4 specific activator of GSK1016790A (1 nM, 10 nM, 20 nM, 50 nM) (i) & (ii). (c) Cell viability test by MTT experiment exhibited inhibition of cell proliferation for melanoma A2058 cells but not for primary epidermal melanocytes by treatment of either DMSO (control) or 2-APB (50 *μ*M, 100 *μ*M, 150 *μ*M, 200 *μ*M, 400 *μ*M) (i) & (ii). (d) Measurement of calcium imaging in GSK1016790A application with 2 nM concentration in A375 melanoma cells was carried out, and intracellular calcium that dramatically enhanced was observed and calcium signal receded after a blocker of ruthenium red was applied (i). A representative single cell (ii) recording by time course showed calcium influx fluctuation before and after GSK1016790A application and channel blocker was added (iii), (n = 12, p < 0.01). (e) Measurement of calcium imaging showed 2-APB could induce calcium influx which could be blocked by TRP channel blocker of ruthenium red in A2058 cells (i), (n = 12, p < 0.01). When TRPV1 agonist of capsaicin was applied, there was no apparent calcium signal observed in A2058 melanoma cells (ii), (n = 19).

Due to the significant upregulation of TRPV4 which has been detected in melanoma A375 cells, GSK1016790A, a selective activator of TRPV4, has been applied to A375 cells as well as melanocytes. Microscopic imaging showed that the proliferation of A375 cells was inhibited after GSK1016790A application but not for melanocytes (Figures 2(a)(i) & 2(a)(ii)). In addition, proliferation was quantified with MTT assay of GSK1016790A (1 nM-50 nM) for 24 h, and GSK1016790A prominently inhibited cell proliferation of A375 cells but with no effect on melanocytes (Figure 2(b), IC_50_ = 10 nM). Meanwhile, 2-APB, an activator of TRPV2 (also activates TRPV1 and TRPV3 but does not affect TRPV4), clearly attenuated the proliferation of A2058 melanoma cells (Figure 2(c), IC_50_ = 150 *μ*M).

To determine whether TRPV4 ion channel functionally mediates the viability of melanoma A375 cells, the activities of TRPV4 then have been investigated. Calcium influx via GSK1016790A application to melanoma A375 cell line was observed by using calcium imaging approach. Assessment by intracellular calcium signals with addition of 2 nM GSK1016790A clearly showed significant increase of calcium signal compared with negative control one by DMSO application, and this calcium influx was clearly attenuated when a TRP channel blocker, ruthenium red was applied (Figures 2(d)(i), 2(d)(ii), and 2(d)(iii), (n = 12, p < 0.01)). These data demonstrated that functional TRPV4 expression could suppress proliferation of human melanoma A375 cells but not for primary epidermal melanocytes.

Calcium imaging assay also identified that TRPV2 has functional activation by 2-APB and channel could be blocked by ruthenium red (Figure 2(e)(i), (n = 12, p < 0.01)). By comparing functional TRPV2 and TRPV1 in A2058 cells affected by its different expression, TRPV1 agonist (capsaicin, 10 *μ*M) was applied, but no apparent calcium signal was detected by calcium imaging approach (Figure 2(e)(ii), (n = 19)). This may be due to very much low expression of TRPV1 ion channel in A2058 cells which could not dominate calcium influx after channel activation.

To further prove that TRPV2 ion channel was modulated by 2-APB in melanoma cells, TRPV2 was knockdown by using shRNA to produce RNA interference in A2058 cells (Figure S2a). As shown in Figure S2, apparent change was hardly observed by treatment of 2-APB in cell viability (Figure S2b). These data suggested that 2-APB targeted TRPV2 ion channel in melanoma A2058 cells and may be implicated in cell fate.

### 3.3. Activation of TRPV2 Channel Promoted Necrosis for A2058 Melanoma Cells

**Figure 3:**
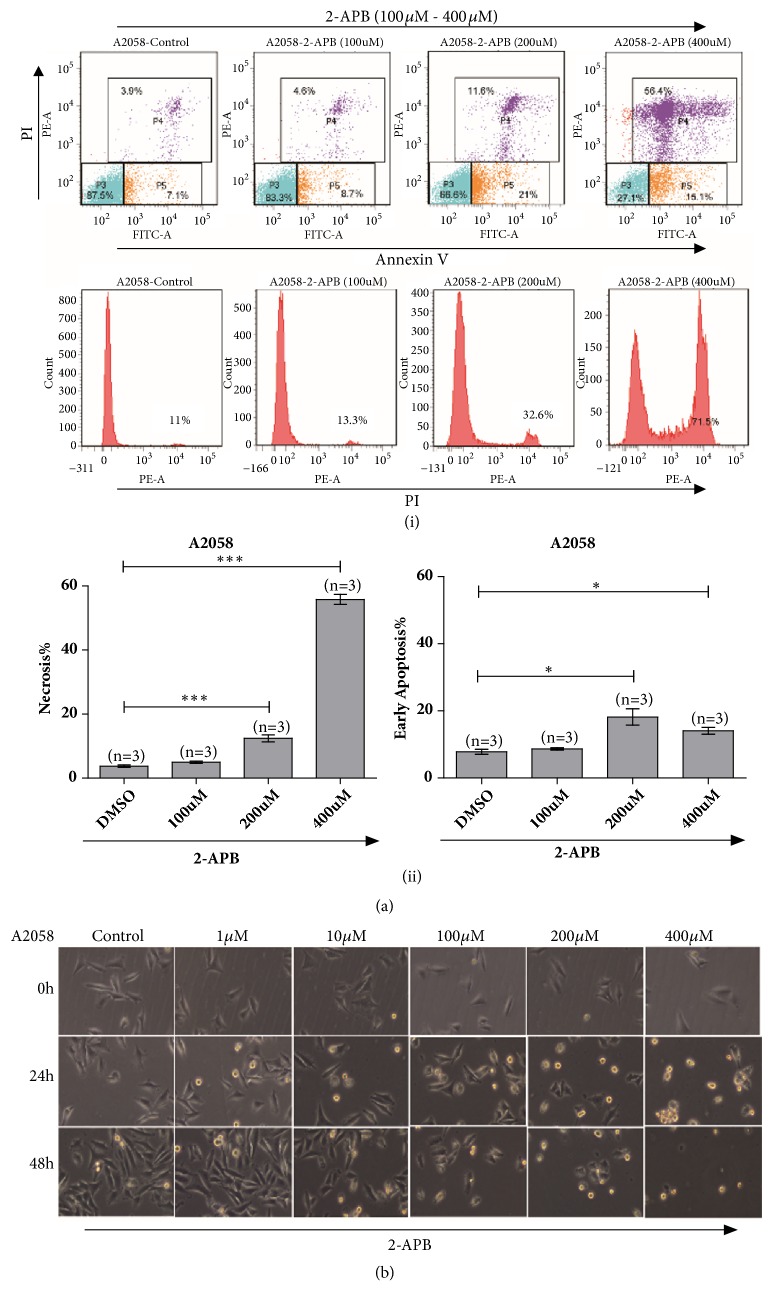
TRPV2 activation dominated A2058 melanoma cells undergoing necrosis. (a) Flow cytometry analysis via FITC-Annexin V and PI staining showed A2058 melanoma cells with 2-APB (100 *μ*M, 200 *μ*M, and 400 *μ*M) treatment undergoing necrosis and enhanced apoptosis (i) & (ii). (b) Morphology measurement by microscopic imaging for A2058 melanoma cells with 2-APB (1 *μ*M, 10 *μ*M, 100 *μ*M, 200 *μ*M, and 400 *μ*M) treatment exhibited prominent necrotic and apoptotic cells.

As activation of TRPV2 could inhibit cell viability of A2058 melanoma cells, the function of TRPV2 in A2058 cells requires further investigation. Flow cytometry analysis for either cell cycle or cell apoptosis has been assessed, respectively. After treatment with 2-APB (100 *μ*M - 400 *μ*M) for 24 h, cell cycle changed negligibly (data not shown), while there was significant increase from 3.9% to 56.4% in the necrotic and late apoptotic cells. Meanwhile, the number of early apoptotic cells was also increased from 7.1% to 15.1% (Figures 3(a)(i) & 3(a)(ii)).

Moreover, microscopic imaging clearly showed that 2-APB could inhibit proliferation of A2058 melanoma cells, and cell swelling and loss of plasma membrane integrity were observed (Figure 3(b)). Taken together, these results indicated that activation of TRPV2 by 2-APB could induce necrosis and apoptosis but does not affect cell cycle for A2058 melanoma cells.

### 3.4. Repression of TRPV4 Abolished Agonist Mediated Signaling Pathway

**Figure 4:**
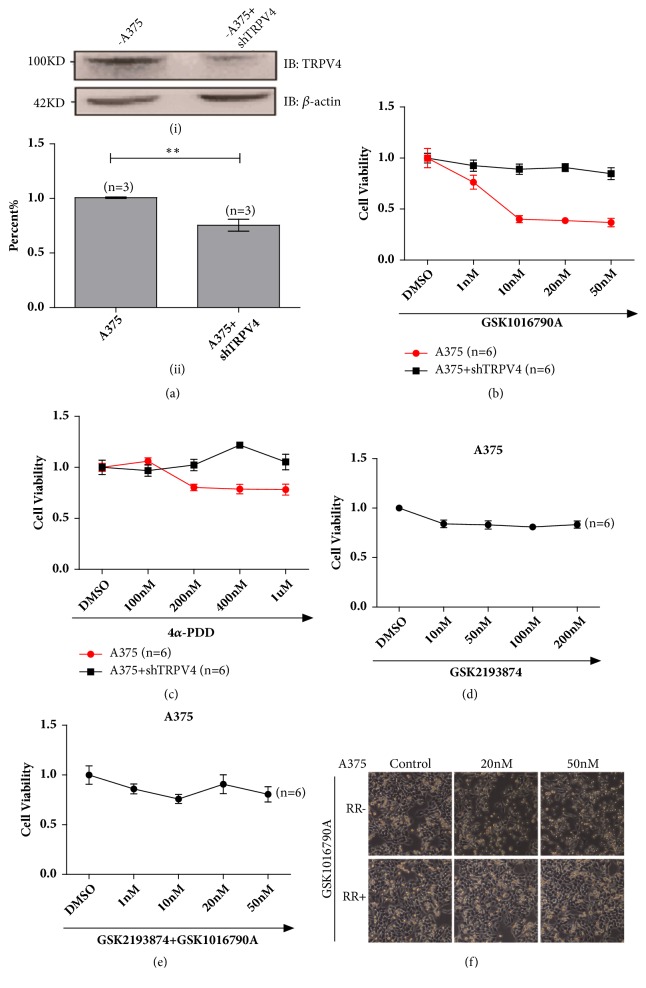
Silencing TRPV4 ion channel suppressed GSK1016790A mediating signals. (a) A representative western blot indicated the successful knockdown of TRPV4 protein by shRNA (i) & (ii). (b) Loss of TRPV4 fully suppressed the inhibition of proliferation for the treatment of GSK1016790A (1 nM, 10 nM, 20 nM, and 50 nM) to A375 melanoma cells. (c) Suppression of TRPV4 attenuated the inhibition of proliferation for the treatment of 4*α*-PDD (100 nM, 200 nM, 400 nM, and 1 *μ*M) in A375 melanoma cells. (d) Inhibition of TRPV4 by GSK2193874 (10 nM, 50 nM, 100 nM and 200 nM) did not affect melanoma cell proliferation. (e) Treatment with GSK1016790A (1 nM, 10 nM, 20 nM and 50 nM) and GSK2193874 (100 nM) did not affect cell viability of melanoma. (f) Pretreatment of ruthenium red fully suppressed the inhibition of proliferation for the application of GSK1016790A with 20 nM and 50 nM to A375 melanoma cells. All tests were performed in at least three independent experiments.

To further prove that functional TRPV4 ion channel mediated melanoma A375 cell viability, TRPV4 was knockdown by using shRNA to produce RNA interference in A375 cells (Figure 4(a)). With TRPV4 knockdown, no significant change was observed in cell viability after GSK1016790A or alternative TRPV4 agonist of 4*α*-PDD for treatment (Figures 4(b) and 4(c)). These results suggested that deficient of TRPV4 could abolish the inhibition of cell proliferation induced by GSK1016790A and 4*α*-PDD.

Moreover, pretreatment of GSK2193874, a selective inhibitor of TRPV4, clearly abrogated the inhibition induced by GSK1016790A (Figures 4(d) and 4(e)). Meanwhile, ruthenium red was also used for pretreatment to suppress TRPV4 function, and similar results were obtained in A375 cells treated with 20 nM and 50 nM GSK1016790A for 24 h (Figure 4(f)). Together, our data demonstrated that TRPV4 channel was functionally involved in cell viability for A375 melanoma cells.

### 3.5. Pharmacological Activation of TRPV4 Induced Apoptosis of A375 Melanoma Cells

**Figure 5:**
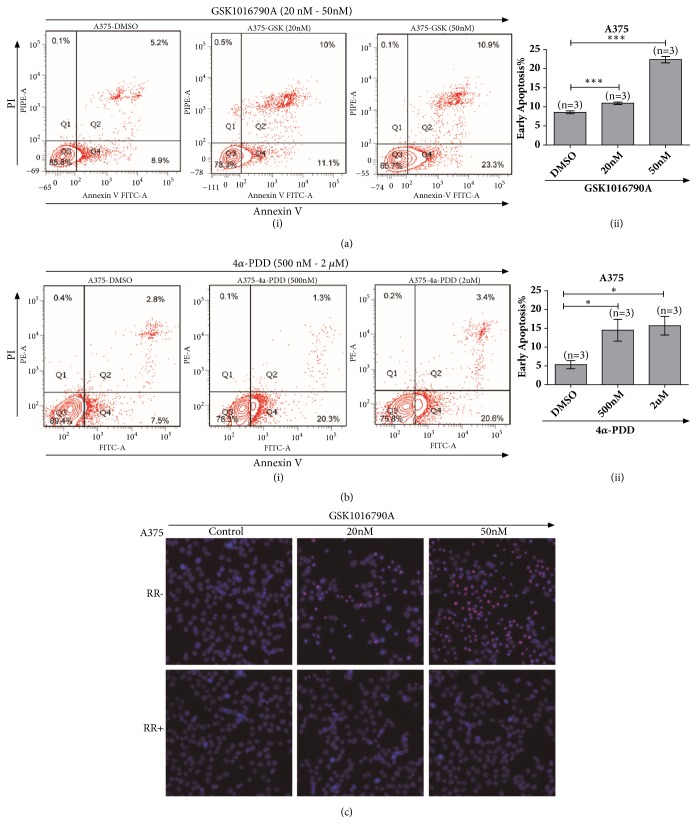
GSK1016790A application induced apoptosis of human A375 melanoma cells. (a) Flow cytometry test via FITC-Annexin V and PI staining showed increasing apoptotic signals by treatment with GSK1016790A to A375 melanoma cells (i) & (ii). (b) Flow cytometry analysis via FITC-Annexin V and PI staining for 4*α*-PDD treatment exhibited enhancing apoptotic cells in melanoma A375 cells (i) & (ii). (c) Pretreatment of ruthenium red prominently attenuated apoptotic cells for the application of GSK1016790A with 20 nM and 50 nM to A375 melanoma cells; cells were stained with Hochest33342 and PI. All tests were performed in at least three independent experiments.

Given that pharmacological activation of TRPV4 could inhibit cell viability, to assess the lethality of GSK1016790A to melanoma cell lines, A375 cells were treated with GSK1016790A at various concentrations for 24 h and then analysed by flow cytometry with FITC conjugated Annexin V and PI. As illustrated in Figure 5, 50 nM GSK1016790A prominently increased the number of apoptotic cells from 8.9% to 23.3% (Figure 5(a)). This data implied that pharmacological application of GSK1017690A could induce apoptosis of A375 cells by activation of TRPV4 channel. Meanwhile, 4*α*-PDD, an agonist of TRPV4 channel, was also applied (500 nM, 2 *μ*M) and the data exhibited similar apoptosis induction from 7.5% to 20.6% in A375 cells (Figure 5(b)).

We also examined cell apoptosis by using microscopy imaging with Hochest33342 and PI staining. Treatment with GSK1016790A clearly enhanced the chromatin condenses in A375 cells. Consistently, after application of channel blocker of ruthenium red, cell viability and chromatin have been observed protected from GSK1016790A addition (20 nM and 50 nM) for 24 h in A375 cells (Figure 5(c)). Together, our data indicated that pharmacological activation of TRPV4 ion channel could induce cell apoptosis of human melanoma.

### 3.6. Functional TRPV4 Channel Involved in Mediating AKT Signaling Pathway via Enhancing AKT Phosphorylation

**Figure 6:**
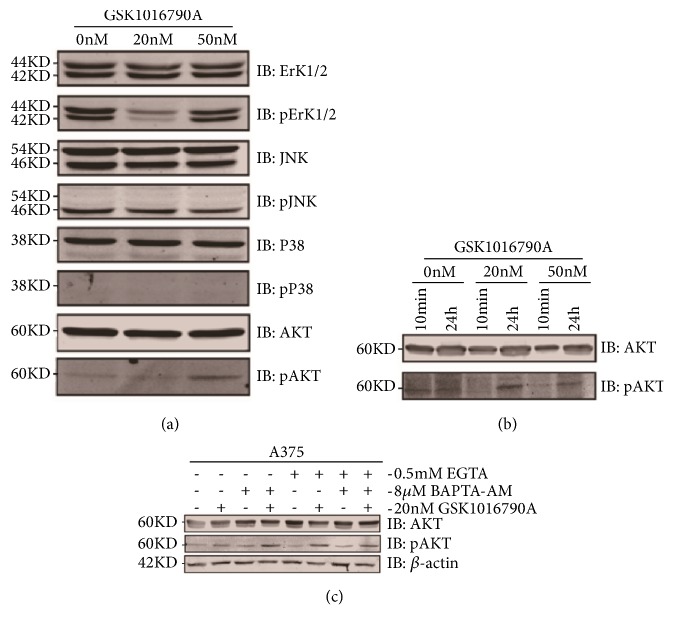
TRPV4 functionally mediated melanoma cells apoptosis via AKT pathway and was influenced by calcium. (a) Western blot analysis of MAPK as well as AKT signals was collected for activation of TRPV4 in A375 melanoma cells. Cells were treated by GSK1016790A with 20 nM and 50 nM for 24 h and lysates were then probed with the indicated antibodies, *β*-actin was used as a loading control, and pAKT was observed upregulated. (b) Assessments of pAKT changes triggered by GSK1016790A with 20 nM and 50 nM within 10 min as well as 24 h in melanoma A375 cells. (c) Influences of calcium ion on GSK1016790A-induced, TRPV4-mediated signal transduction. A375 cells were untreated or pretreated with EGTA or BAPTA-AM for 1 h before being stimulated with GSK1016790A. All tests were performed in at least three independent experiments.

How TRPV4 mediated apoptosis of A375 cells by its activation is unclear. Since AKT and MAPK signaling pathways are commonly associated with cell apoptosis, we hypothesized that TRPV4 may mediate cell apoptosis of melanoma via AKT or MAPK signaling pathway. Phosphorylation of AKT was significantly greater in GSK1016790A application in A375 cells compared to control one (Figures 6(a) and 6(b)), while ErK/JNK/P38 of MAPK signal pathways were not influenced in application of GSK1016790A (Figure 6(a)). These data suggested that the increased phosphorylation and activation of AKT pathway (Figure 6(b)) may be involved in GSK1016790A inducing cell apoptosis of melanoma via functional TRPV4 ion channel.

### 3.7. Calcium Signaling Mediated by TRPV4 Facilitated Apoptosis via Phosphorylation of AKT in A375 Melanoma Cells

TRPV4 is a nonselective cationic channel with high permeability of calcium. Activation of TRPV4 ion channel would trigger calcium influx. As an important intracellular second messenger, calcium is involved in a bunch of physiological and pathological processes via ion channels [33, 34]. Is calcium involved in the regulation of functional TRPV4 in melanoma? Calcium chelator of EGTA (ethylene glycol tetra-acetic acid) and BAPTA-AM were applied, and the AKT signals were collected from calcium/calcium free medium incubation cells of GSK1016790A addition. In the absence of extracellular calcium chelator of EGTA or intracellular calcium chelator of BAPTA-AM, 20 nM GSK1016790A addition could enhance the phosphorylation of AKT, while depletion of extracellular calcium by EGTA, GSK1016790A even potentiated the phosphorylation of AKT signal greater in A375 cells. Meanwhile, in the presence of intracellular calcium chelator of BAPTA-AM, GSK1016790A seemed to have similar effects as EGTA application on the phosphorylation of AKT pathway. Indeed, when there was depletion of both extra- and intracellular calcium, AKT phosphorylation induced by GSK1016790A was attenuated in A375 cells. These data indicated that calcium was involved in regulation of AKT phosphorylation following GSK1016790A treatment since EGTA or BAPTA-AM pretreatment clearly enhanced GSK1016790A induced rise in AKT phosphorylation via TRPV4 ion channel (Figure 6(c)). Thus, AKT functioned as downstream effectors for calcium signaling mediated by TRPV4 in human A375 melanoma cells.

## 4. Discussion

Thermo-TRPs are not only localized in sensory neurons as polymodal cellular sensors but also functionally expressed in a plethora of nonneuronal cell types involved in regulating various physiological and pathophysiological functions [35–40]. In TRPM subfamily, TRPM2 and TRPM8 were implicated in the regulation of melanocytic behaviors. TRPM2 was capable of inducing melanoma apoptosis and necrosis. TRPM8 could mediate agonist-induced melanoma cell death [41]. In the present study, six thermo-TRPs such as TRPV1/2/3/4, TRPA1, and TRPM8 were examined. Our data showed that there was discordance between protein and gene expression, such as TRPV2 in human melanoma cells. Previous studies have revealed that discrepancy between protein and mRNA expression levels was widely variable. For example, mRNA and protein discordance has been reported to vary from 32% [42] to 83.5% [43] in the LNCaP prostate cancer cell line. There are several factors that could result in the discordance. According to our results, thermo-TRPs are low abundant membrane proteins, and actual biological differences between transcript and protein abundance may affect the correlation between gene and protein expression levels just as previous studies indicated [44].

**Figure 7:**
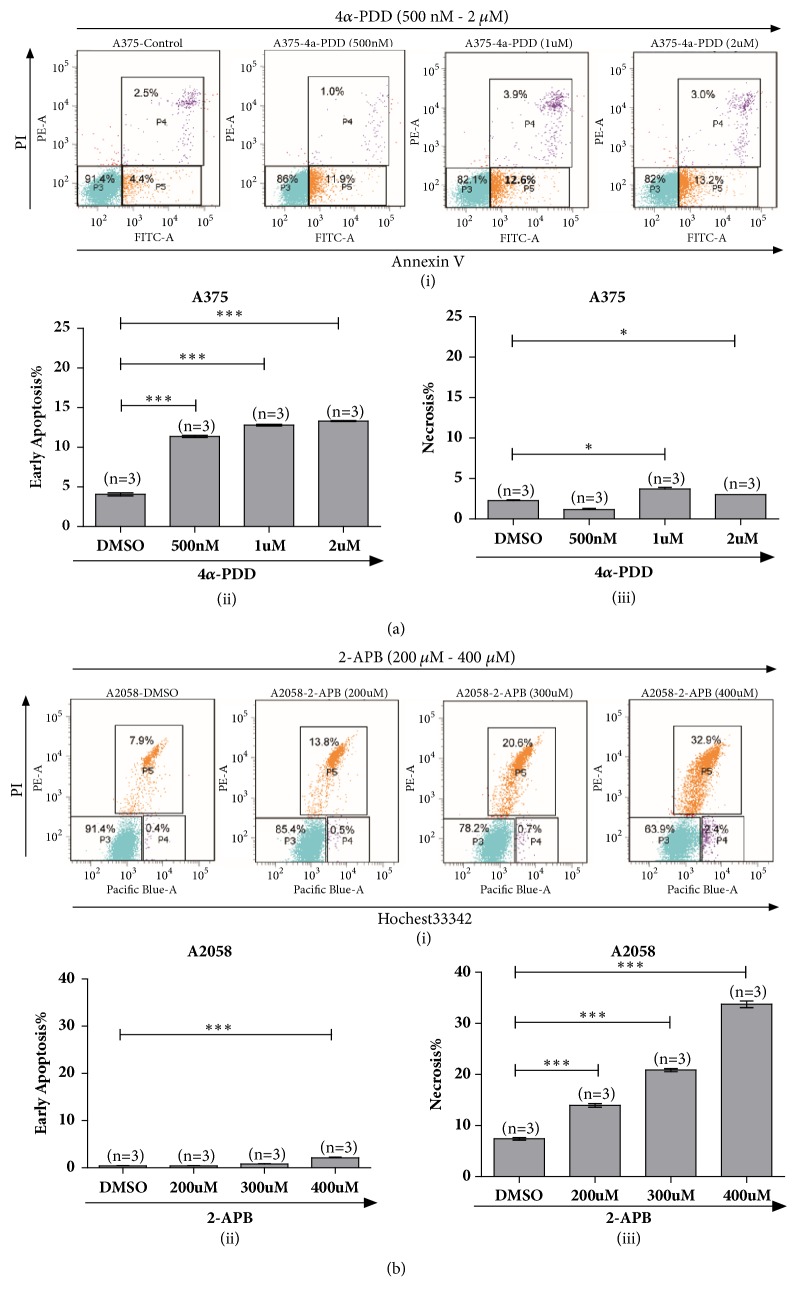
Functional TRPVs influenced melanoma cell fate in different profiles. (a) Flow cytometry assessment of 4*α*-PDD triggered apoptosis (i) of melanoma A375 cells via staining with FITC - Annexin V and PI. Activation of TRPV4 by 4*α*-PDD induced human melanoma A375 cells undergoing early apoptosis (ii). A slight degree of necrotic cells was observed as well (iii). (b) Flow cytometry assessment of 2-APB triggered cell death of melanoma A2058 cells via staining with Hochest33342 and PI. Activation of TRPV2 by 2-APB in melanoma A2058 cells underwent late apoptosis as well as necrosis (i). 2-APB application induced a slight degree of early apoptotic cells (ii) and dominated cells undergoing necrosis and late apoptosis (iii).

Recent studies have tried to elucidate the distributions and roles of several thermo-TRPs in cancer. Fusi and colleagues investigated a subset of TRP ion channels including TRPV1/2/3/4 and TRPA1 in human nonmelanoma skin cancer. They found downregulation of TRPV4 could act as an early biomarker of skin carcinogenesis [12]. Tsavaler et al. reported that TRPM8 was upregulated in prostate cancer when compared with normal prostate epithelial cells. In addition, TRPM8 also can be detected in tumors of breast, colon, lung, and skin but not in the corresponding normal human tissues [45]. Our present study identified upregulation of TRPV4 and activation of this ion channel could induce apparent intracellular calcium signaling in regulation of cell viability and apoptosis. Notably, cells underwent early apoptosis after TRPV4 activation by 4*α*-PDD (Figure 7(a)) as well as GSK1016790A. Meanwhile, in melanoma A2058 cells, pharmacological activation of TRPV2 with 2-APB could inhibit cell proliferation and promote cell apoptosis as well. Interestingly, 2-APB application dominated A2058 cells undergoing necrosis and late apoptosis other than early apoptosis both via FITC-Annexin V/PI staining and Hochest33342/PI staining (Figure 7(b)), which was clearly different from the activation of TRPV4 in melanoma A375 cells. Our data further indicated that activation of thermo-TRPV2/4 modulated distinct cellular behaviors in human melanoma cells.

Early studies suggested that TRPV1 was involved in skin cancer [13, 46]. Inhibition of TRPV1 by antagonist of AMG9810 could promote mouse skin tumorigenesis mediated through EGFR/AKT/ mTOR signaling pathway [46]. Based on our examination, it is not the case for this study. Independent study reported that treatment of a TRPV1 agonist of capsaicin could efficiently reduce proliferation and induce obvious apoptosis of renal carcinoma cells through mediating caspases activation via P38 and JNK within MAPK pathway [47]. Other independent study revealed that TRPV6 might mediate growth factor signaling to induce PI3K-PKD1-AKT cascade via calcium in human colon cancer [48]. In this regard, AKT and MAPK signaling pathways were examined in the present study. And our data highlighted that AKT signaling regulated cell fate of A375 melanoma cells during TRPV4 activation via enhancing AKT phosphorylation.

Characteristics of cells including neoplastic human melanoma cells are determined by Ca^2+^ dependent cellular process. Intracellular calcium is predominantly regulated by TRPs [33]. Thus, calcium involvement was assessed in the present study. Our results demonstrated that calcium involved in TRPV4 mediation of AKT phosphorylation. Notably, depletion of either extracellular or intracellular calcium could clearly potentiate AKT phosphorylation induced by GSK1016790A. These observations indicated that TRPV4 served as distinct cellular linker between calcium ions and calcium signaling pathway and thus was expected to dynamically modulate AKT phosphorylation process. Further studies are needed to fully understand the cooperative modulation among calcium changes and related downstream signaling of AKT.

## 5. Conclusions

Taken together, here we have demonstrated that TRPV2 and TRPV4 were distributed in human melanoma A2058 and A375 cells as well as melanocytes. Pharmacological activation of TRPV4 could inhibit cell proliferation and induce cell apoptosis of A375 cells. Furthermore, the activation of TRPV4-AKT pathway with calcium signaling involvement drove antitumor effect in A375 cells. Our data suggested that TRPV4 ion channel therefore may serve as a potential clinical therapeutic strategy in human melanoma.

## Figures and Tables

**Table 1:** Primer sequences and reference number of human TRP-specific primers used in digital PCRs.

Channel	Forward primer	Reverse primer	NM-number
TRPV1	ATCGCCCGTCCTGGTATCA	CCTCCTCCGAGTCACCCTT	NM_080705.3

TRPV2	TCTTCCTTTTCGGCTTCGC	CCCTCGTCCTCCTGTCCCT	NM_016113.4

TRPV3	GCTGCGTGGAGGAGTTGG	CAGGTCTTCCCCGTGTCG	NM_001258205.1

TRPV4	TGGAGTCACATAAGCCAACGC	GGCAAATCCCAGACACTACAGA	NM_021625.4

TRPA1	GTTTGGCAGTTGGCGACA	GGATACACGATGGTGGATTTCT	NM_007332.2

TRPM8	GCAATGCCATCTCCTACGC	TGAAGGTCAGCAGACTCCCA	NM_024080.4

*β*-actin	TGGCATCCACGAAACTACCTT	TCGTCATACTCCTGCTTGCTG	NM_001101.3

## Data Availability

The data used to support the findings of this study are available from the corresponding author upon request.

## Abbreviations

GSK: GSK1016790A
PI: Propidium iodide
Pro: Probenecid
TRP: Transient Receptor Potential
TRPV: Transient Receptor Potential Vanilloid
RR: Ruthenium red.

## Supplementary Materials

Supplementary Materials See Figures S1 and S2 in the Supplementary Materials for comprehensive image analysis.
