# Supplementary material for: Mechanism for Regulation of Melanoma Cell Death via Activation of Thermo-TRPV4 and TRPV2
Source: J Oncol. 2019 Feb 7;2019:7362875. doi: 10.1155/2019/7362875 (PMC6383420; doi:10.1155/2019/7362875)
Supplement: Supplementary Materials — See Figures S1 and S2 in the Supplementary Materials for comprehensive image analysis. [file 7362875.f1.pdf]

Supplementary Materials

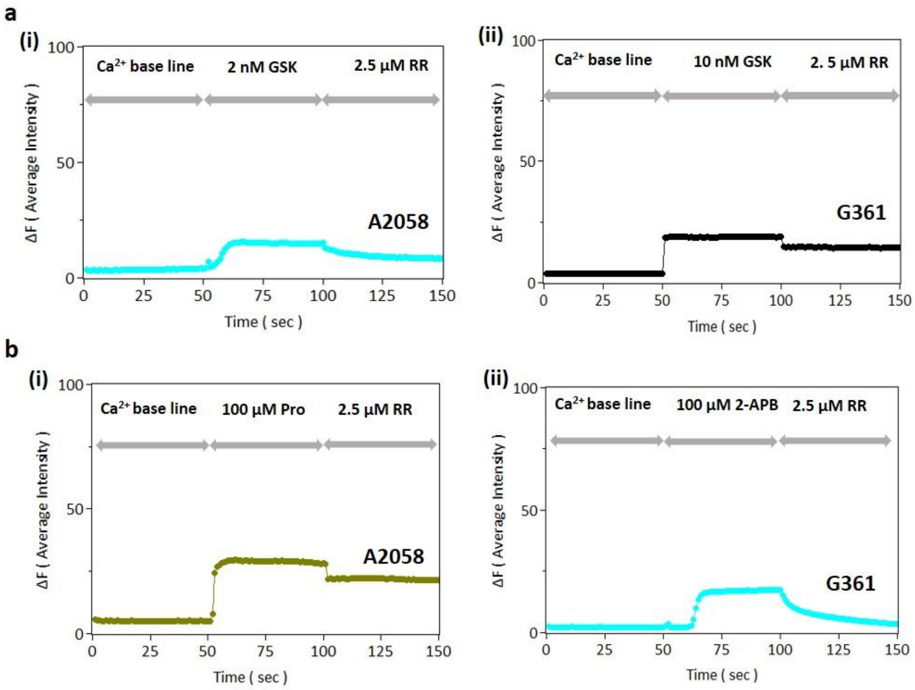

Figure S1: Functional TRPs distributed in human melanoma cells. (a) Measurement of calcium imaging in GSK1016790A application with 2 nM concentration in A2058 (i), (n =25) and G361 (ii), ($n = 12$, $p < 0.05$) melanoma cells, and intracellular calcium slightly enhanced and calcium signal receded after a blocker of ruthenium red was applied. (b) Measurement of calcium imaging showed probenecid could induce calcium influx which could be blocked by TRP channel blocker of ruthenium red in A2058 cells (i), ($n = 23$, $p < 0.01$). When 2-APB was applied to G361 cells, there was slight increase in calcium influx observed and ruthenium red also made calcium influx receded (ii), ($n = 13$, $p < 0.05$).

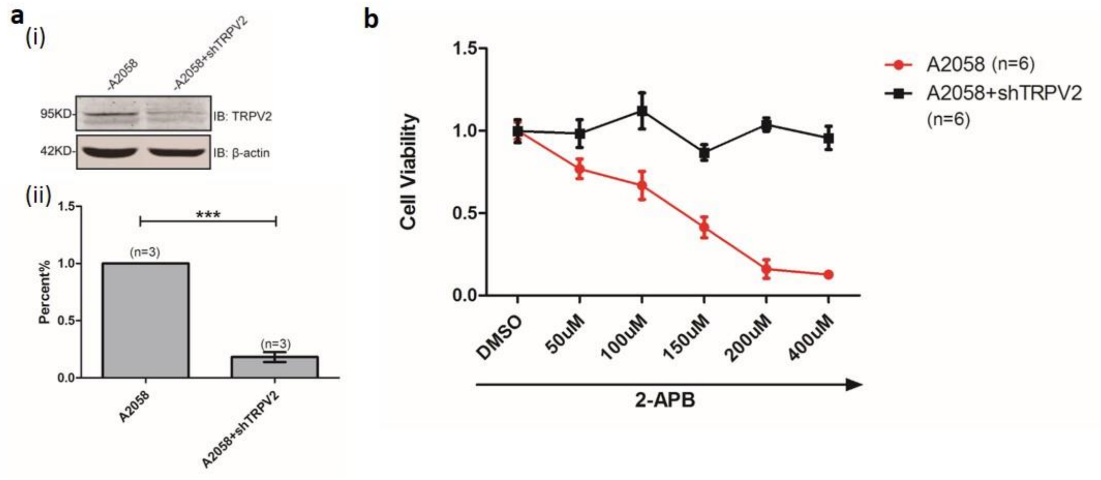

Figure S2: Knockdown of TRPV2 abolished 2-APB mediating signals. (a) A representative western blot indicated the successful knockdown of TRPV2 protein by shRNA (i) & (ii). (b) Loss of TRPV2 fully suppressed the inhibition of proliferation for the treatment of 2-APB (50 μM, 100 μM, 150 μM, 200 μM, 400 μM) to A2058 melanoma cells.
